# Smear Layer Removal and Microhardness Alteration Potential of a Naturally Occurring Antioxidant – An In Vitro Study

**DOI:** 10.7759/cureus.5241

**Published:** 2019-07-25

**Authors:** Arasappan Rajakumaran, Hasini Ramesh, Rupa Ashok, Lakshmi Balaji, Arathi Ganesh

**Affiliations:** 1 Conservative Dentistry and Endodontics, Sri Ramachandra Institute of Higher Education and Research, Chennai, IND

**Keywords:** smear layer, dentin microhardness, scanning electron microscopy, n-acetyl cysteine (nac), ethylenediaminetetraacetic acid (edta)

## Abstract

Introduction: It is well-known in the field of endodontics that the presence of a smear layer in the root canals can harbor bacteria and limit the penetration of irrigating solutions and intracanal medications into the dentinal tubules which, in turn, causes the failure of endodontic treatment. Removing the entire smear layer throughout the root canal is essential for the success of endodontic treatment and these chemical agents that facilitate the removal of the smear layer are called chelating agents. Ethylenediaminetetraacetic acid (EDTA), being the most widely used chelating agent, brings about increased reduction in the microhardness of the root dentin, thereby making it friable.

N-acetyl cysteine (NAC) is naturally occurring antioxidant that has various beneficial properties for the human being. Several studies have been done in determining the antimicrobial efficacy of NAC against various endodontic pathogens and concluded NAC to be advantageous. The chelating property of NAC has been utilized in heavy metal detoxification, where it binds to the metal ion and removes them from the human system. However, this chelating property has not been explored in the field of endodontics.

Aim: This study was aimed to compare the ability of N-acetyl cysteine with the conventional chelating agent in the removal of the smear layer and in altering the microhardness of root dentin.

Materials and Methodology: A total of 84 single-rooted human mandibular premolars with relatively similar dimension and morphology, freshly extracted with closed apices, were collected from adult patients. The crowns of all specimens were cut transversally at the cementoenamel junction (CEJ) with the double-faced diamond disc at low speed, with water coolant, to obtain a 12 mm root length. The root canals were randomly divided into three equal groups according to the final irrigation solutions: Group I: 17% EDTA, Group II: 20% NAC, and Group III: distilled water (control). They were then randomly divided into two parts: scanning electron microscope (SEM) analysis for the extent of smear layer removal and microhardness evaluation using the Vicker’s hardness test.

Results: The smear layer removal ability of EDTA and NAC were more effective in the coronal and middle thirds of the root canal. However, both groups showed less smear layer removal in the apical region. Specimens treated with distilled water showed the least reduction in the smear layer throughout the length of the root canals. Regarding the evaluation of microhardness, both EDTA and NAC had a significant reduction in root dentin microhardness. However, the percentage of dentin microhardness reduction was significantly more in the EDTA group (p < 0.05) than N-acetyl cysteine in the coronal, middle, and apical third of the root canals.

Conclusion: The chelating property of NAC is equally effective to that of EDTA in the smear layer from the root canal, and it induced a significantly lesser reduction in microhardness of root dentin than EDTA.

## Introduction

Root canal morphology is complex by nature and dealing with such complexity is challenging as it hampers the ability to achieve thorough disinfection of the pulp cavity. Chemo-mechanical debridement of the pulp space with the aid of instruments and irrigating solutions determines the outcome of endodontic therapy. Root canal instrumentation produces a layer of organic and inorganic material called the smear layer that may also contain bacteria and their by-products [1]. It is essential to remove the entire smear layer in the root dentin for a successful endodontic treatment and the chemical agents which aid in removing the smear layer are called chelating agents. Some of these chelating agents are capable of causing alterations in the chemical composition of dentin. Any change in the calcium to phosphorous ratio may, in turn, change the microhardness, permeability, and solubility characteristics of dentin and may also adversely affect the sealing ability and adhesion of dental materials [2]. The conventional chelating agents bring about an increased reduction in microhardness of root dentin, thereby affecting the integrity of the tooth structure.

Chelators are stable complexes of metal ions with organic substances as a result of ring-shaped bonds [1]. Chelating agents decalcify the dentin by combining with the calcium ions of the tooth structure, unlike acids, which dissolve the inorganic structure of dentin by their low pH. The decalcifying effect of chelating agents depends largely on the application time, solution pH, and concentrations [3]. Although many chelating agents have been tested over the past four decades, no single best agent has yet been identified for use in smear layer removal.

EDTA is generally accepted as the most effective chelating agent in endodontic therapy. It is used to remove the smear layer and to prepare the dentinal walls for better adhesion of filling materials. The disodium salt of EDTA at a 17% concentration and neutral pH is widely preferred for root canal treatment. The recommended protocol for effective removal of both organic and inorganic components of the smear layer is sodium hypochlorite, followed by EDTA or citric acid. However, EDTA also has the potential for causing excessive dentinal erosion and can cause a moderate degree of irritation [4].

N-acetyl cysteine (NAC) is a naturally occurring antioxidant, which is the acetylated form of the amino acid cysteine [5]. In clinical medicine, it has been used in the detoxification of heavy metals, such as mercury and lead [6]. Many studies have explored the antioxidant and antibacterial properties of NAC. Surprisingly, the possible effectiveness of NAC in removing the smear layer and altering the microhardness of root dentin as a chelating agent in dentistry has not been extensively perused, although this property has been used greatly used in clinical medicine.

Hence, the aim of this study was to compare the ability of NAC with a conventional chelating agent in the removal of the smear layer and in altering the microhardness of the root dentin.

## Materials and methods

A total of 84 freshly extracted, single-rooted human mandibular premolars, with relatively similar dimension and morphology, were collected from adult patients. Each tooth was radiographed to confirm the presence of a single canal. Teeth with previous root caries, cracks, curved canals, endodontic treatment, internal resorption, or calcification were excluded. The selected teeth were cleaned and decontaminated by immersion in a 5.25% sodium hypochlorite solution for 30 minutes and stored in a sterile saline solution at room temperature [3]. The crowns of all specimens were cut transversally at the cementoenamel junction (CEJ) with the double-faced diamond disc (#4217) (DFS-Diamon GmbH, Riedenburg, Germany) at low speed, with water coolant, to obtain a 12 mm root length.

The root canals were randomly divided into three equal groups according to the final irrigation solutions. Access to the root canals were obtained using a round burr. The root canals of the teeth in all the three groups were irrigated with distilled water and shaping was done using Protaper Next® rotary files (Dentsply Maillefer, Ballaigues, Switzerland) till size X2. After instrumentation, final irrigation was done with one of the experimental solutions. A 30-gauge needle was used for irrigation and was inserted within 1 - 2 mm from the root apex. The experimental solutions used in each group were as follows: Group I: 17% EDTA, Group II: 20% NAC, Group III: distilled water (control).

The specimens were randomly allocated for scanning electron microscope (SEM) analysis and microhardness evaluation.

Preparation of NAC experimental solution

NAC solution (Sisco Research Laboratories, Mumbai, India), at a concentration of 200 mg/ml, was freshly prepared by dissolving 0.2 gm in 1 ml of sterile distilled water according to Quah et al. [7].

Evaluation of smear layer removal

A total of 48 roots were subjected for evaluation of the smear layer removal potential of the chelating agents. Each specimen was flushed with a volume of 5 ml of experimental irrigant solution for a period of one minute. After final irrigation, each root canal was flushed and copiously irrigated with 10 ml distilled water and dried with absorbent paper points (Dia Pro-T, Diadent, Canada). Two longitudinal grooves were prepared on the buccal and lingual surfaces of each root using a diamond disc and each root was then split longitudinally into two halves using a mallet and a stainless-steel chisel.

The roots sections were coded and mounted on metallic stubs. Specimens were sputter-coated with gold to render the surface electrically conductive and observed with SEM (JEOL Co., Tokyo, Japan). SEM photomicrographs of the specimens were taken at a 1000x magnification at the coronal third, middle third, and apical third of the root canals [8].

The photomicrographs were saved and analyzed for the presence or absence of the smear layer by means of the scoring system proposed by Hulsmann et al. [9]. The scoring system consisted of Score 1: no smear layer, dentinal tubules open; Score 2: small amount of smear layer, some dentinal tubules open; Score 3: homogeneous smear layer covering the root canal wall, only a few dentinal tubules open; Score 4: complete root canal wall covered by a homogeneous smear layer, no open dentinal tubules; and Score 5: heavy, inhomogeneous smear layer covering the complete root canal wall.

The final result for each section of the canals was obtained by calculating the mean of the scores of each of the photographs. The data were tabulated for statistical analysis using the Statistical Package for Social Sciences (SPSS) (IBM SPSS Statistics, Armonk, NY) computer software. A descriptive analysis was computed as the frequency of each score for each tested group.

Evaluation of microhardness

A total of 36 roots were subjected for evaluation of microhardness. For longitudinal sectioning of the root, longitudinal grooves were made on the buccal and lingual external root surface. These grooves were made using a double-faced diamond disc at low speed with care not to penetrate the root canals. Roots were then split into two halves which resulted in 72 specimens. Each prepared root half was horizontally embedded in an acrylic block, such that their dentin was exposed. Each root half was labeled on the acrylic block for indentation. The test surface was grounded with 400, 500, and 600 grit silicon carbide paper in a polishing machine (Metmech, Chennai, India) followed by hand polishing with a felt disc embedded with aluminum oxide paste.

For testing the microhardness of the dentin surface, the specimens were randomly divided into the three experimental groups (n = 24) and specimens were treated with an experimental irrigation solution for five minutes.

Microhardness was measured for each sample before and after application of different irrigating solutions. Microhardness testing was carried out using the Vickers microhardness tester (HDNS Kelly instruments, Shanghai, China) with a Vickers diamond indenter and a 40x objective lens to obtain a pre-treatment record for each individual half. Mean baseline Vickers hardness number (VHN) was calculated for each specimen at the cervical, middle, and apical third. The indentation was made on the dentin surface approximately 0.5 mm from the root canal space. Each measurement was carried out using a 200 gm load for 10 seconds, oriented perpendicularly to the root surface. The diagonal lengths of indentations were measured by a built-in scaled micrometer and measurements were converted into Vickers numbers.

In order to prevent the dilution of the irrigants before the experiment, excess fluid was removed from the canal surface with sterile paper points. All experimental specimens were then flushed with 30 ml of sterile saline [7]. Specimens were blotted dry with sterile paper points. The microhardness was measured for canal dentine surface after irrigation in the same way for each sample as a baseline measurement to record the post-treatment VHN. The change in microhardness was calculated as the difference between baseline values and post-application values after immersion in the tested irrigants.

The data were collected and tabulated for statistical analysis using the SPSS computer software. Inferential statistical analysis was done using one-way analysis of variance (ANOVA) to detect the difference between tested groups.

## Results

Evaluation of smear layer removal

**Figure 1:**
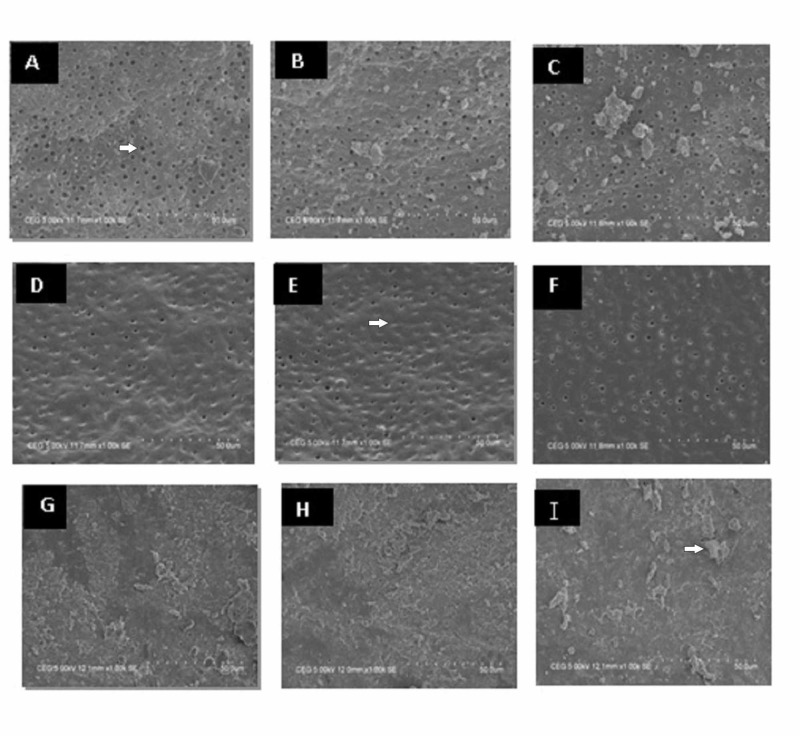
Scanning electron microscope (SEM) photomicrograph of the experimental groups A) Group I, coronal third (arrow denotes open dentinal tubules); B) Group I, middle third; C) Group I, apical third; D) Group II, coronal third; E) Group II, middle third (arrow denotes occluded dentinal tubules); F) Group II, apical third; G) Group III, coronal third; H) Group III, middle third; I) Group III, apical third (arrow denotes clumps of smear layer)

The smear layer removal ability of Groups I and II were more effective in the coronal and middle third of the root canal. However, both groups showed less smear layer removal in the apical region. Specimens treated with saline showed the least reduction in the smear layer in the coronal, middle, and apical thirds of the root canals. In Group I, the coronal third showed a Score 3 in all the specimens (Figure 1A), while for Group II, the coronal third showed a Score 2 in 18 specimens and a Score 3 in six specimens (Figure 1D). However, the middle and apical thirds showed a Score 3 in both Groups I and II for all the specimens (Figure 1B-F). In Group III, the coronal, middle, and apical thirds showed a Score 4 in all specimens (Figure 1G-I).

Evaluation of microhardness

All irrigating solutions decreased the microhardness of the root canal dentin surface compared to the baseline measurements. Group I exhibited the highest reduction in root canal dentin microhardness, followed by Group II. Group III showed the least reduction in root dentin microhardness. The baseline measurements of microhardness of the root canal dentin surface showed no significant difference between the tested groups. The baseline microhardness value for each individual root sample was compared with its post-treatment. The hardness of radicular dentin varied at the cervical, middle, and apical levels, as shown in Table 1.

**Table 1:** Pre and Post-treatment Values of Vickers Microhardness Measurement p < 0.05

Region	Group I - Ethylenediaminetetraacetic acid (EDTA)		Group II - N-acetyl cysteine (NAC)		Group III – Control	
	Mean	Mean	Mean	Mean	Mean	Mean
	Pre	Post	Pre	Post	Pre	Post
Coronal	51.15 ± 1.1	46.05 ± 2.3	52.95 ± 1.23	48.85 ± 2.1	48.2 ± 0.54	47.25 ± 1.05
Middle	49.5 ± 2.15	42.65 ± 1.91	47.95 ± 1.56	45.1 ± 2.13	46.8 ± 0.45	45.65 ± 1.24
Apical	42.95 ± 0.59	38.25 ± 1.23	46.6 ± 0.39	43.5 ± 1.20	43.45 ± 0.35	43.1 ± 1.52

Statistical analysis using ANOVA showed that Groups I and II had a significant reduction in root dentin microhardness (p < 0.05) in the coronal, middle, and apical third of the root canal, as shown in Table 2. Group III showed the least change in microhardness between the baseline and post-treatment measurements.

**Table 2:** Reduction in Microhardness Values SD: standard deviation

Group	Mean % Reduction ± SD
Group I	10.5 ± 0.2831
Group II	6.763 ± 0.5112
Group III	1.75 ± 0.4537

## Discussion

During root canal instrumentation, there can be the formation of a smear layer, which can limit the penetration of the irrigating solution and inter-appointment medications into dentinal tubules [10]. Chemicals used to remove the smear layer may lead to alterations in root dentin and changes in its chemical and physical properties. These chemicals also bring about a reduction in microhardness of the root dentin, which can facilitate the instrumentation throughout the canal but may also weaken the root structure. Microhardness determination can provide indirect evidence for losing or gaining any mineral substance in the dental hard tissues [11].

The most widely used chelating solution is EDTA, which acts by forming stable complexes with calcium present on the smear layer and removes it [3]. EDTA has a strong demineralizing effect, causes enlargement of dentinal tubules, softening of the dentin, and denaturation of the collagen fibers. It has the potential for causing excessive dentinal erosion [4]. The search for a chelating agent that was more efficient and biocompatible than the EDTA has resulted in various solutions being researched over the last few years [12-15].

NAC is a thiol-containing antioxidant that possesses an antibacterial property against endodontic pathogens, such as Enterococcus faecalis [7, 16-17], Actinomyces naeslundii, Lactobacillus salivarius, and Streptococcus mutans [17]. The active moiety is the thiol (-SH) site, which plays a role in free radical scavenging and destruction of the intermolecular or intramolecular disulfide bonds in proteins [18]. Quah et al. found the antibacterial property of NAC was unaffected by the presence of dentin, which can be considered as an advantage of the drug [7]. NAC has been widely perused in the field of clinical medicine for its anti-inflammatory property in chronic obstructive pulmonary disease and chronic bronchitis [19-20] and also as a chelator to remove methylmercury toxins from the body [6]. Hence, the present study was designed to test the chelating property of NAC by smear layer removal and the reduction in microhardness of the root dentin when compared to EDTA, the conventional chelating solution.

Most irrigation materials come up short in removing the smear layer, particularly in the apical third of the canals [14]. The entire canal length was utilized to test the efficacy of the solutions in all segments of the root, including the apical third. Root canals in this investigation were prepared with the Protaper Next rotary system as the use of the rotary instruments creates a significant amount of smear layer. Syringe irrigation can control both the volume and depth of the syringe penetration, which results in the flow of the irrigant to the apical region of the canal system. On this basis, all irrigations were done using a 30-gauge needle, as recommended by Plotino et al. [21]. Scanning electron microscopy has been used to determine the effectiveness of various irrigants to remove the smear layer as it allows an examination of morphologic details of the surfaces of the prepared root canal [22].

The microhardness of dentin may vary considerably between teeth; thus, in the present study, the microhardness measurement was performed for each sample at baseline and after treatment with the irrigation solutions to establish a reasonable evaluation for the effect of the irrigant solutions on the dentin surface. Post-treatment indentations were performed on each sample at the same areas that were at symmetrical constant points of the baseline for both sides of the root canal to make an evaluation of the tested irrigant [13]. The microhardness measurement was performed in three points at the coronal, middle, and apical third of the root canal dentin. The mean Vickers hardness number (VHN) was calculated for each specimen. The microhardness of the dentin depends on the tubular density, which varies from one area to another on the root dentin surface. Therefore, the current study design followed Pashley et al. [23], who stated that the tubular density affects microhardness - as the tubular density increases, the dentin microhardness decreases. Distilled water was used initially as an irrigant because it has no effect on the dentin surface; thus, it was not considered as a variable which might affect the results [24]. This was followed by the application of endodontic irrigation solutions on the root canal dentin surface for five minutes, according to the papers published by De-Deus et al. [12] and Sayin et al. [25]. The selection of the Vickers microhardness tester over the Knoop hardness tester was due to the suitability and practicality of the Vickers test for evaluating surface changes of deeper dental hard tissues. The Knoop hardness tester is used for superficial dentin at 0.1 mm rather than for deep dentin [26].

A possible limitation of the current study was that the experiments were performed at room temperature and not body temperature. Additionally, the volume of the irrigant in a root canal clinically was small compared with the immersing root dentin in irrigating solutions. However, standardized circumstances for all study groups allowed for comparable results. The present study revealed that both irrigation solutions decreased dentin microhardness with the exception of the distilled water. This finding is in accordance with Sayin et al. [25], who concluded that significant alteration in dentin hardness after the irrigation treatment indicated potent direct effects of these chemical solutions on the components of dentin structure.

The chelating action of the EDTA solution induces an adverse softening potential on the calcified components of the dentin, and subsequently, a reduction in the microhardness was expected. Studies have shown that EDTA facilitates chelation of the inorganic portion of dentin and, consequently, makes it much weaker than normal [26-27]. In the present study, EDTA induced the greatest reduction in root dentin microhardness, possibly because of its strong demineralizing effect owing to its high acidity and the ability to calcify root dentin, with the most calcium and phosphorus extracted during its application compared with NAC [28]. N-acetyl cysteine induced a lower reduction in root dentin microhardness than EDTA and this could be owing to its softer chelating nature and lower depth of demineralization.

## Conclusions

The results of the current study substantiated the chelating property of NAC to be equally effective to that of EDTA in passively removing the smear layer from the root canal and it induced a significantly lesser reduction in the microhardness of the root dentin than EDTA. Hence, with further studies, NAC can be explored to serve the triple purpose of an active irrigant, a potent intracanal medicament, and a chelating agent in root canal therapy.
